# Tunneling nanotubes (TNT) mediate long-range gap junctional communication: *Implications for HIV cell to cell spread*

**DOI:** 10.1038/s41598-017-16600-1

**Published:** 2017-11-30

**Authors:** George Okafo, Lisa Prevedel, Eliseo Eugenin

**Affiliations:** 1 GlaxoSmithKline, Stevenage, SG1 2NY UK; 2 Public Health Research Institute (PHRI), Newark, NJ USA; 3 Department of Microbiology, Biochemistry and Molecular Genetics, Rutgers New Jersey Medical School, Rutgers the State University of NJ, Newark, NJ USA

## Abstract

Cell-to-cell communication is essen for the development of multicellular systems and is coordinated by soluble factors, exosomes, gap junction (GJ) channels, and the recently described tunneling nanotubes (TNTs). We and others have demonstrated that TNT-like structures are mostly present during pathogenic conditions, including HIV infection. However, the nature, function, and communication properties of TNTs are still poorly understood. In this manuscript, we demonstrate that TNTs induced by HIV infection have functional GJs at the ends of their membrane extensions and that TNTs mediate long-range GJ communication during HIV infection. Blocking or reducing GJ communication during HIV infection resulted in aberrant TNT cell-to-cell contact, compromising HIV spread and replication. Thus, TNTs and associated GJs are required for the efficient cell-to-cell communication and viral spread. Our data indicate that targeting TNTs/GJs may provide new therapeutic opportunities for the treatment of HIV.

## Introduction

Tunneling nanotubes (TNTs) and gap junctions (GJs) are the only two communication systems that allow direct exchange of cytoplasmic factors between connected cells^1,2^. Both TNTs, which are specialized membrane projections, and GJs, which are formed by the docking of connexin-43 (Cx43) containing channels in the membranes of interacting cells, participate in key biological processes, including development, signaling, and immune response, but are also involved in the pathogenesis of several diseases, including HIV^3–5^. Currently, it is assumed that the major differences between TNTs and GJs are the distances required to establish plasma membrane contacts and the potential size of the cargos transferred between connected cells. Specifically, TNTs allow long-range communication, whereas GJs mediate shorter range cell-to-cell interactions. Also, while GJs only allow the exchange of small molecules (up to 1.2 kDa), including second messengers and small peptides^6,7^, TNTs are able to transfer both small molecules and mediate the exchange of larger organelles and vesicles^5,8–10^. Although both TNTs and GJs are known to exist and mediate important cell-to-cell interactions, whether and how these two systems interact with each other has not been explored.

In this study, we present evidence of direct physical interplay between TNTs and GJs during HIV infection. We show that TNTs induced by HIV infection contain functional GJ channels at their ends and that TNT-gap junctional communication is required for efficient viral replication and cell-to-cell spread. Our finding, identify TNTs and GJs as critical mediators of HIV infectivity but also during reactivation when the virus needs to use host systems to amplify infection.

## Materials and Methods

### Materials

All reagents were purchased from Sigma (St. Louis, MO), except in the places that are indicated otherwise. HIV_ADA_ was from the NIH AIDS Research and Reference Reagent Program (Germantown, MD). RPMI, fetal bovine serum (FBS), penicillin/streptomycin (P/S) and trypsin-EDTA were from Thermofisher (Grand Island, NY). Phalloidin-conjugate to Texas red and anti-fade with DAPI were obtained from Thermo Fisher (Eugene, OR). The HIV-p24 antibody was from Genetex (Irvine, CA). Purified mouse IgG_2B_ and IgG_1_ myeloma protein were from Cappel Pharmaceuticals, Inc. (Aurora, OH). All protocols were evaluated and approved by Rutgers University. Human tissues were part of an ongoing research protocol approved by Rutgers University (IRB protocols Pro2012001303 and Pro20140000794).

### HIV-infection of primary cultures of monocytes/macrophages

Blood was obtained from healthy volunteers (NY Blood Center), and PBMC were isolated by Ficoll-Paque (GE Healthcare, Uppsala, Sweden). After PBMC isolation, monocytes/macrophages were allowed to adhere to glass for 3 days. Cells were cultured in RPMI-1640 supplemented with 10% FCS, 5% human AB serum, 10 mM HEPES, P/S and 10 ng/ml M-CSF (Peprotech, Rocky Hill, NJ). After 6–7 days in culture, the cells were infected with HIV (20 ng/ml HIV-p24/1 × 10^6^ cells). After 24 h of exposure to the virus, cells were washed extensively to eliminate the unbound virus before addition of fresh medium and then supernatants were collected every day to assess viral replication by HIV-p24 ELISA.

### Immunofluorescence

Human monocyte-derived macrophages, HIV-infected and uninfected, were grown on glass coverslips, fixed and permeabilized in 70% ethanol for 20 min at −20 °C. Cells were incubated in blocking solution for 30 min at room temperature and then in primary antibody (anti-connexin43 F(ab)’_2_ fragments and anti-HIV-p24 or isotype controls: both 1:2,500 or 1:50) overnight at 4 °C. Cells were washed several times with PBS at room temperature and incubated with phalloidin conjugated to Texas Red to identify actin filaments and/or the appropriate secondary antibody conjugated to FITC (Sigma, St. Louis, MO) for 1 h at room temperature, followed by another wash in PBS for 1 h. Coverslips were then mounted using anti-fade reagent with DAPI, and cells were examined by confocal microscopy using an A1 Nikon confocal microscope with spectral detection (Tokyo, Japan).

### Dye coupling

Gap junctional communication was tested by observing the intercellular transfer of Lucifer Yellow (5% w/v LY dissolved in 150 mM LiCl) microinjected through glass microelectrodes by brief overcompensation of the negative capacitance circuit in the amplifier until the impaled cell was brightly fluorescent. After dye injection, cells were observed for 1–4 min to determine whether dye transfer occurred as described previously^11^. The incidence of dye coupling was calculated by dividing the number of injected cells showing dye transfer to more than one neighboring cell by the total number of cells injected in each experiment multiplied by one hundred. Also, the diffusion of the dye from the site of injection into the TNT communicated cells was calculated. Fluorescence was quantified using an imaging program, NIS Elements (Tokyo, Japan). Dye coupling was observed in an inverted Z1 microscope with incubation systems (Zeiss, Germany).

### Electron microscopy

Cells were fixed for 30 min at RT using 4% paraformaldehyde, 2% glutaraldehyde, buffered with 0.1 M sodium cacodylate. Cells were dried with hexamethyldisilazane until fully dry under a fume hood. The cells were analyzed using a Zeiss SUPRA 40 field emission scanning electron microscopy (SEM) and placed on a fitted mould for the holder. The holder was calibrated, and cells were imaged at various magnifications as indicated with accelerating voltage of 3 kV. For transmission electron microscopy (TEM), a JEOL1200EX electron microscope was used. This protocol allowed the structure of the TNTs and filopodia and cell shape to be maintained.

### ELISA for HIV-p24

Quantification of the release of HIV-p24 was determined by ELISA according to the manufacturer’s protocols (Perkin Elmer, Boston, MA).

### Statistical Analysis

Student’s one-tailed paired t-test was used to compare the numbers and length of processes and the HIV-p24 values. A value of p < 0.05 was considered significant.

## Results

### Characterization of TNT-like structures in uninfected and HIV-infected macrophages

Currently, most TNT studies use live cell imaging or confocal microscopy techniques to identify and quantify TNT numbers and other characteristics such as length, branching, protein content, cargo transported, the speed of vesicular transport, and cell-to-cell signaling^1–5,10,12–25^. However, the main limitation of these methodologies is the diffraction limit of the equipment, which has a range of around 250 nm (x-axis) × 800 nm (y-axis)^2,26^. Moreover, the Y-axis resolution is not optimal for a cellular process of around 50 to 700 nm in diameter, depending on the cell and imaging system used^8,10,27^. To address these deficiencies, we examined TNTs using scanning (SEM) and transmission (TEM) electron microscopy.

**Figure 1:**
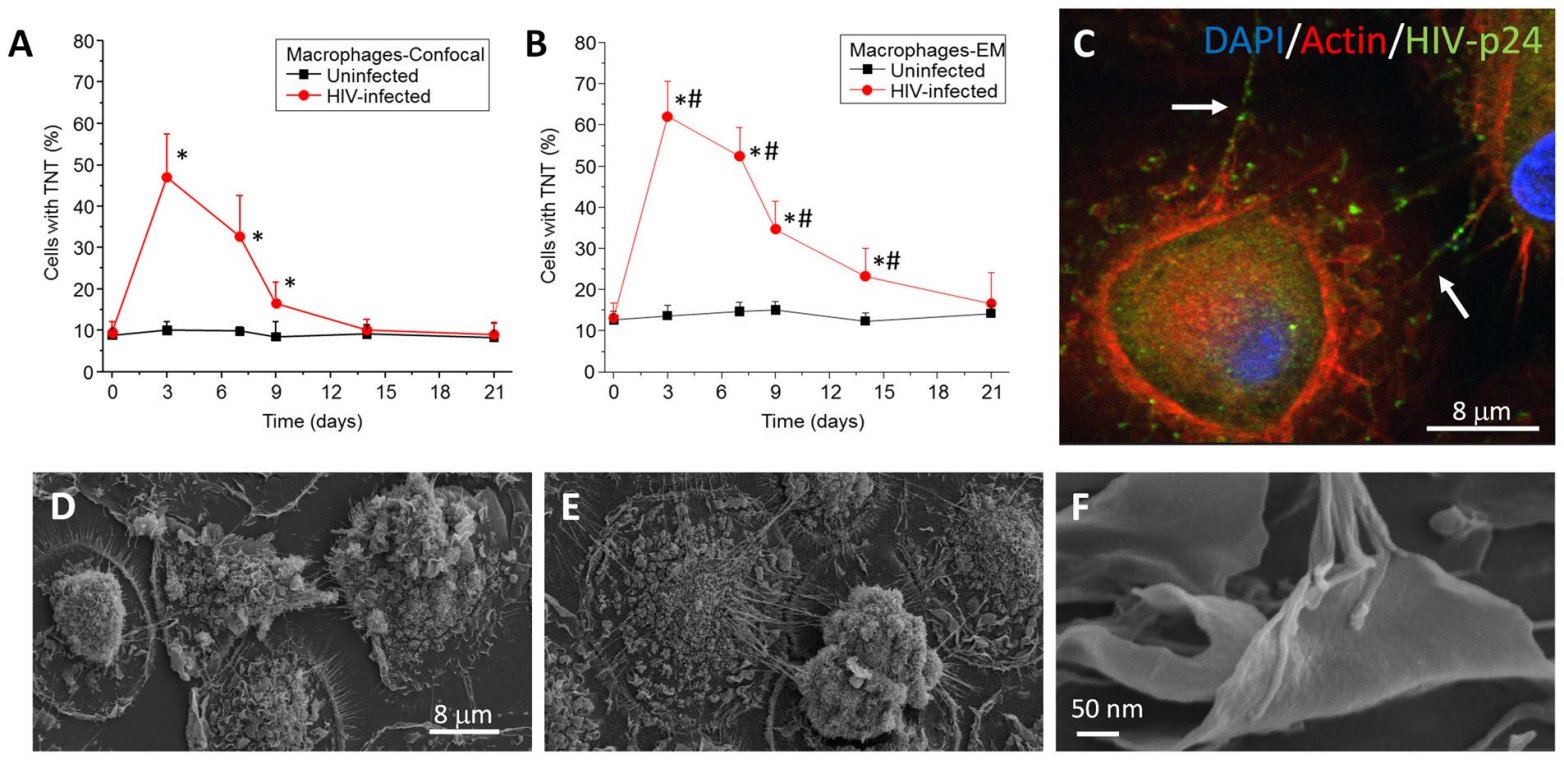
HIV infection of human macrophages results in increased numbers of TNTs. Cultures of human macrophages were exposed to HIV_ADA_ for 24 h and then washed extensively to eliminate unbound virus. Media was collected every day to assay for secreted HIV-p24 by ELISA. In parallel, at different time points, cells were fixed for confocal microscopy or SEM. (**A**) Time course of formation of TNT or numbers of cells expressing TNTs using confocal microscopy. Uninfected cells contained low numbers of TNT (black line). However, HIV infection induced the formation of TNT per cell as well as the numbers of cells with TNT (red line). *p ≤ 0.0005 as compared to uninfected conditions, n = 23. (**B**) Time course of formation of TNT or numbers of cells expressing TNTs using SEM. Quantification of cells with TNT indicates that SEM is able to detect 1–20% higher numbers of cells with TNT than confocal microscopy. *p ≤ 0.0008 as compared to uninfected conditions. ^#^p ≤ 0.003 as compared to the values in (**A**) in the presence of HIV, n = 5. (**C**) Representative image of macrophages infected with HIV after 3 days’ post infection and stained with DAPI, Actin, and HIV-p24. HIV-p24 (green staining) is spread by TNTs (white arrows). No background or nonspecific staining was detected using isotype-matched irrelevant antibodies (data not shown). (**D**) Representative image of 3 days of culture of uninfected macrophages analyzed by SEM. (**E**) Representative image of HIV infected cultures after 3 days post infection. (**F**) High magnification of the end of the TNT process in HIV infected conditions. n = 5.

To corroborate our published data^2,28^, we characterized and quantified TNTs in human primary macrophages using confocal microscopy. Uninfected and HIV-infected cultures were fixed at different time points (0, 3, 7, 9, 14, and 21 days) and analyzed by confocal microscopy using staining for DAPI (to stain nuclei), Phalloidin (to stain actin) and HIV-p24 (to stain an HIV protein) (Fig. 1A). Confocal analysis indicated that uninfected macrophages had fewer TNT-like structures (6.7 ± 4.34% of the cells, Fig. 1A, black line) than infected macrophages (around 40–50% of the cells, Fig. 1A, red line), and this difference was observed through most of the time-course. Hence, under uninfected conditions, TNT-mediated intercellular communication in macrophages is likely to be limited due to low TNT formation. In contrast, under HIV-infected conditions (HIV_ADA_, 20 ng/ml), TNT processes proliferate (Fig. 1A), correlating well with previous publications involving HIV cell-to-cell spread and replication^2,28^. The formation of TNT correlated with the numbers of HIV infected positive cells. After 60–80% of the cells were positive for HIV-p24, TNT formation or stability decreased to control levels, suggesting that TNT are only necessary during the active cell to cell infection process. Using confocal microscopy we were able to detect HIV-p24 (Fig. 1C, green staining) moving through TNTs, but the resolution of the confocal only allowed the identification of a cellular process with undefined characteristics (Fig. 1C, representative picture after 3 days post-HIV infection).

Analysis of the same macrophage cultures by SEM produced results similar to those obtained by confocal microscopy (Fig. 1B). However, the detection levels of TNTs or cells containing TNTs were higher than those detected by confocal microscopy. For example, SEM but not confocal microscopy detected TNTs at 14 days’ post-infection (Fig. 1A,B). This difference is most likely due to improved XY resolution of SEM. Also using SEM, we observed that, while TNTs were minimally expressed in uninfected cultures (Fig. 1D), under HIV-infected conditions TNTs proliferated and that each TNT observed by confocal microscopy contained several cellular processes (Fig. 1E,F). Furthermore, higher magnification of the ends of the TNT processes indicated that the connections between HIV-infected and uninfected macrophages were mostly of the synaptic kind (Fig. 1F). Also, TNTs observed by SEM corresponded to multiple intertwined processes (Fig. 1E,F). Further analysis of macrophage cultures by SEM indicated that HIV-infection did not change the number of filopodium or membrane ruffling (data not shown). Furthermore, we determined that TNTs originated from the membrane of the HIV-infected macrophages to reach the membrane ruffling present on the surface of uninfected macrophages. In conclusion, HIV infection induced the dynamic formation of TNTs, and SEM was used to describe TNT numbers and structures with the level of detail not achieved by previously used imaging methodologies.

### TNT-like structures connecting HIV-infected and uninfected human macrophages contain connexin43 at the tip of the process

Our published data in rodent macrophage/microglia demonstrated that under specific inflammatory conditions, these cells express connexin-43 (Cx43), use GJ communication, and contain TNT-like structures^7,29,30^. Also, we showed that HIV infection, *via* binding of HIV-tat to the Cx43 promoter, induced or maintained the expression of Cx43 to favor HIV infection and associated inflammation^2,31,32^. However, it was unknown where Cx43 was expressed in relation to TNT processes and whether these Cx43 proteins formed functional GJs.

**Figure 2:**
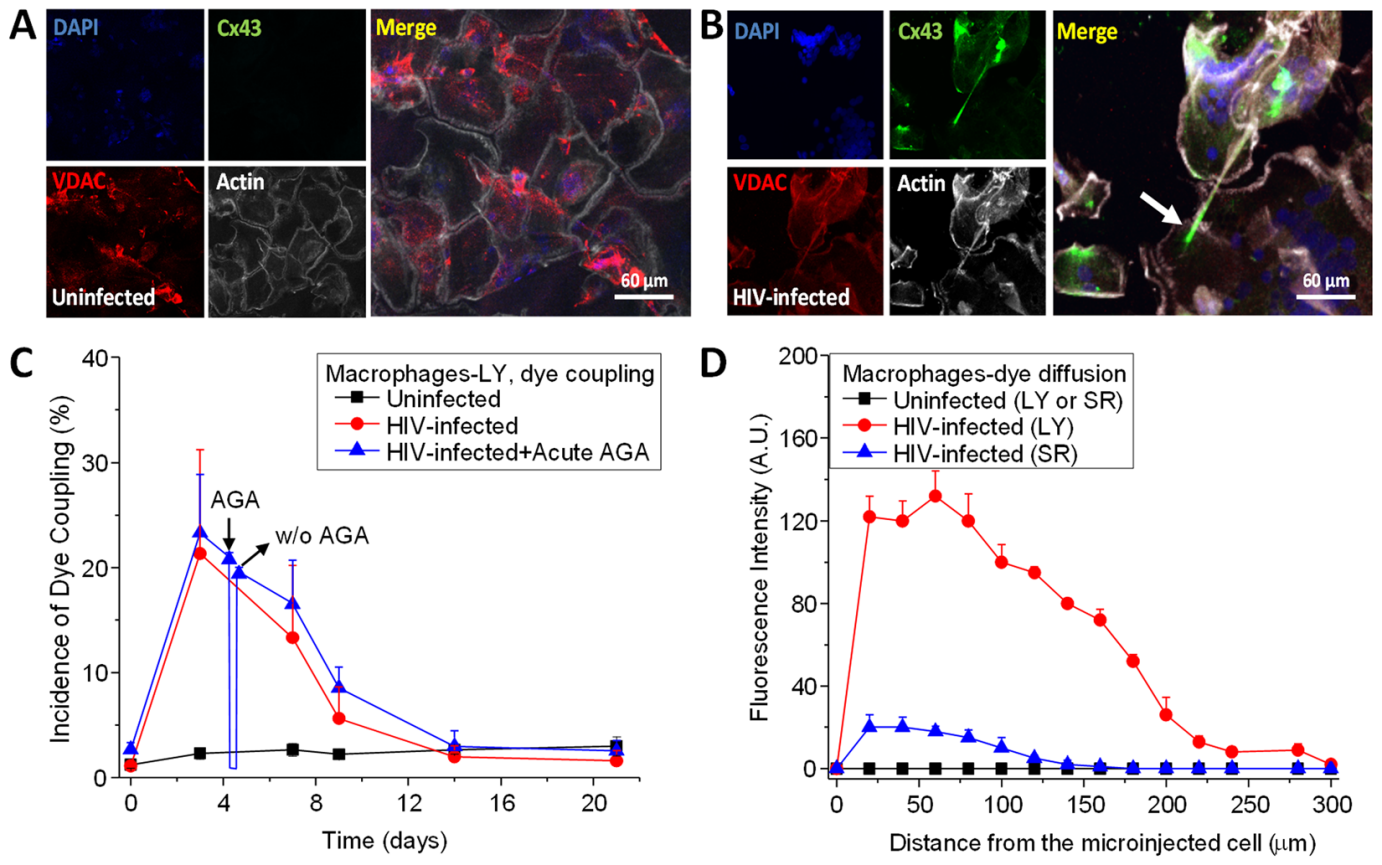
Cx43 is localized at the tip of the TNTs in HIV infected macrophages. Staining for DAPI (nuclear dye, blue staining), Cx43 (green staining), VDAC (mitochondrial marker, red staining), and actin (white staining) in uninfected (**A**) and HIV infected (**B**) cultures of macrophages. In uninfected conditions, no staining for Cx43 was detected at any time point analyzed. (**B**) In HIV infected cultures, Cx43 was expressed and mainly localized at the end of the TNT process (see arrow). (**C**) Microinjection of LY to evaluate dye coupling between TNT connected macrophages. LY microinjection in uninfected cultures shows no dye coupling (black line). HIV infection resulted in significant increase in cell to cell transfer of LY supporting active gap junctional communication during the time that TNTs are formed (red line, compare to Fig. 1). Acute application or washout (W/O) of AGA to reversible block gap junctions indicates that AGA reversible block the dye coupling (blue line). (**D**) Quantification of the spread of LY fluorescence from the microinjected cell into TNT neighboring communicated cells. In uninfected cultures, microinjection of LY or sulforhodamine (SR) did not result in diffusion of the dyes into neighboring cells (black lines). Formation of TNTs by HIV infection resulted in the diffusion of LY and SR into TNT communicated macrophages (red and blue line, respectively).

To examine the Cx43 expression and distribution in TNTs, we stained macrophages under uninfected and HIV-infected conditions during the same time-course as above (Fig. 1) using DAPI (nuclear, blue staining), Cx43 (green staining), VDAC (voltage-dependent anion channel, mitochondrial marker, red staining) and phalloidin (actin, white staining). Uninfected human macrophages did not show any Cx43 expression at any of the time points analyzed (Fig. 2A), which is consistent with our previous publications^29,33^. In HIV-infected conditions, Cx43 expression was induced early, at three days’ post-infection, and remained high throughout the time course analyzed (Fig. 2B). Most Cx43 proteins were localized in an intracellular compartment at the base of the TNT, as well as at the tip of the TNT, with minimal co-localization with VDAC (Fig. 2B). Several groups have reported that functional Cx43 was observed on the surface of the mitochondria^34,35^, but in our studies, no co-localization was observed. Also, the transfer of mitotracker-labeled mitochondria or VDAC positive organelles was not observed between TNT-connected macrophages in uninfected or HIV infected conditions (data not shown), suggesting that Cx43 trafficking was independent of mitochondrial movement. These results suggested that HIV-induced the expression and localization of Cx43 into the TNT synaptic contacts between HIV-infected and uninfected macrophages.

To further evaluate the role of Cx43 expression and localization within TNTs in GJ communication, Lucifer Yellow (LY) was microinjected into TNT-connected macrophages to assess transfer of the dye between these connected macrophages. Microinjection of dye into uninfected macrophages did not result in any dye diffusion between TNT-connected cells at all the times tested (Fig. 2C, black line). In contrast, microinjection of LY into HIV-infected macrophages resulted in the diffusion of LY into the TNT-connected cell, which supported the hypothesis that the Cx43 present at the end of the TNT process contributed to GJ communication (Fig. 2C, red line). This conclusion was supported by the observation that dye coupling induced by HIV infection was sensitive to acute application of the GJ blockers, octanol (500 µM; data not shown) or 18-alpha-glycyrrhetinic acid (AGA, 32 µM) (Fig. 2C, acute AGA, blue line). Washout (w/o) of AGA resulted in partial recovery of the dye coupling which suggested that the effect was specific and reversible (Fig. 2C, blue line, w/o).

Considering the observed low dye coupling, as well as the extremely well-localized expression of Cx43 at the tip of the TNT process, the diffusion of the fluorescent dye into the TNT acceptor cell was quantified to determine the diffusion of the dye as an additional measure of dye coupling (Fig. 2D). No LY or sulforhodamine (SR) diffusion was observed in uninfected cultures at any time point (Fig. 2D, black line). In HIV-infected cultures, the diffusion of LY, but not SR (Fig. 2D, blue line), reached distances up to 200 µm (Fig. 2D, red line), indicating that TNT-GJ coupling was an effective long-range communication mechanism. The diffusion of LY was also susceptible to GJ blockers, AGA or octanol treatment (data not shown). In addition, the application of LY into the extracellular medium to examine the potential opening of hemichannels in response to HIV exposure did not result in LY uptake, which suggested that Cx43 hemichannels were not present on the surface of uninfected or HIV-infected macrophage cultures (data not shown). Thus, these data demonstrated that Cx43 present at the tip of the TNTs mediates functional GJ communication in response to HIV-infection.

### Blocking GJ communication prevented the effective formation of the TNT synaptic contact between HIV-infected and uninfected macrophages

**Figure 3:**
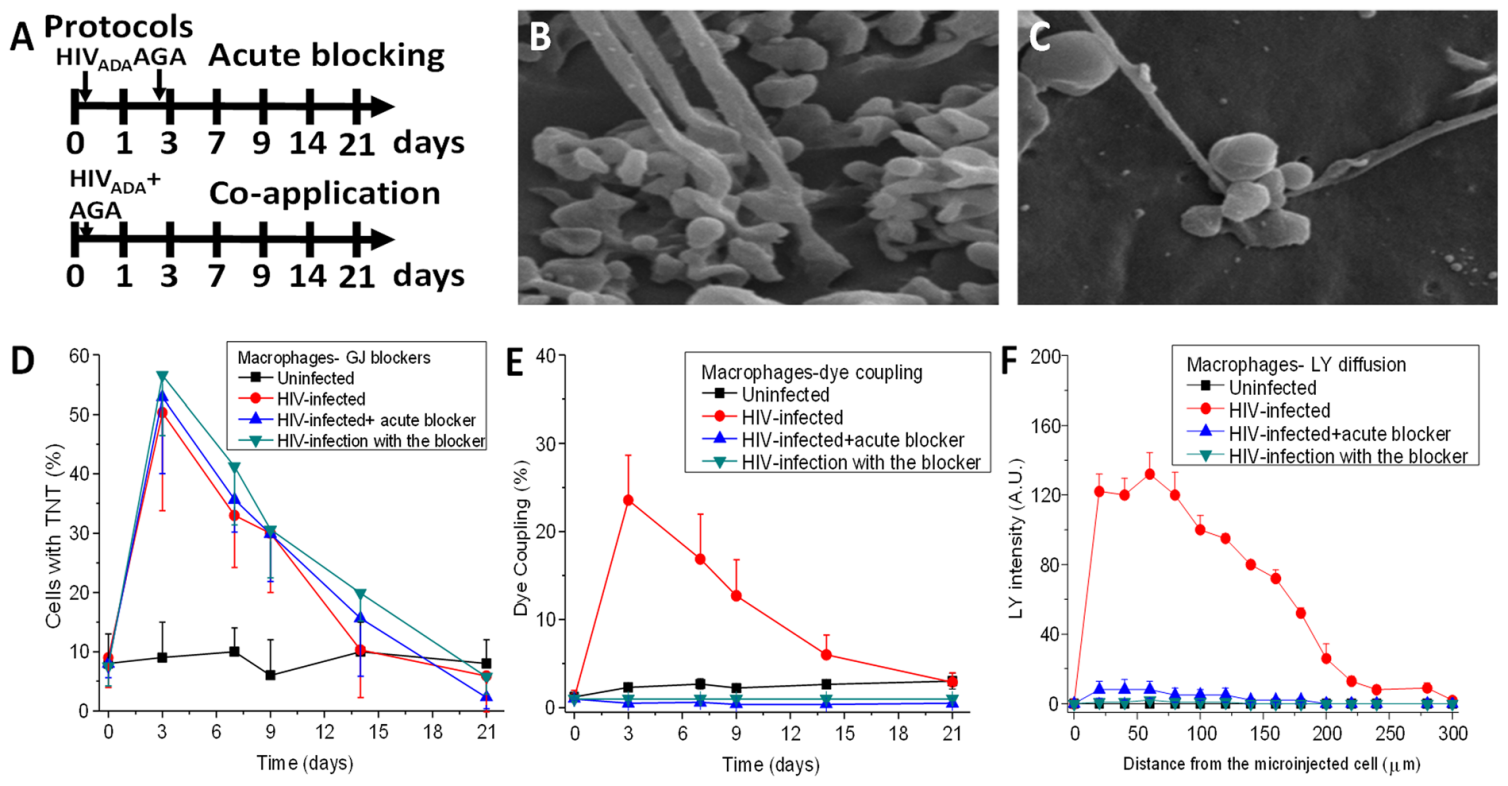
Gap junctions expressed at the tip are not required for the formation of TNT, but are necessary to establish the synaptic contact with the recipient cell. (**A**) A graphic description of both protocols used in our studies. Acute blocking protocol corresponded to HIV infection for 24 h and subsequent application of the gap junction blocker AGA after 3 days of infection. This protocol enables the virus to enter, integrate, and replicate for 3 days (protocol labeled acute blocking). In this case TNTs are formed, before the application of the gap junction blockers. The second protocol corresponds to co-application of HIV plus AGA (labeled co-application). (**B**) Under HIV conditions, a synaptic kind of interaction occurs between the cells forming the TNT and the recipient cell. (**C**) Both protocols using AGA, a GJ blocker, resulted in the aberrant formation of TNT-cell body interactions. The tip of the TNT process shows clear signs of swelling and aberrant cell to cell contact. (**D**) Time course of formation of TNT in uninfected (line with □), HIV infected cultures (line with •), HIV-infection with acute GJ blocking (line with ∇), and HIV-infection with the blocker (co-application, line with ◊). No significant differences in TNT formation were observed in all conditions (p ≥ 0.120, n = 4). (**E**) Time course of dye coupling in uninfected (line with □), HIV infected cultures (line with •), HIV-infection with acute GJ blocking (line with Δ), and HIV-infection with the blocker (co-application, line with ∇). Dye coupling evaluation shown that functional gap junctions at the tip of the TNT are required for effective communication between TNT communicated cells (all treatments were significant, except time point 0 and 21 days’ post infection, p ≤ 0.002, n = 4 as compared to HIV infection alone, red line). (**F**) Time course of LY diffusion from the microinjected cell into the TNT communicated cell in uninfected, HIV infected cultures (red line), HIV-infection with acute GJ blocking, and HIV-infection with the blocker (co-application). LY diffusion demonstrates that lack of proper formation of gap junctions at the tip of TNT compromise effective gap junctional communication (n = 3).

To characterize the role of GJ communication in TNT formation and stability, two different GJ blocking protocols were developed (Fig. 3A). The first protocol consisted of HIV infection as described above and subsequent acute application of AGA (GJ blocker) after 3 days post-infection (Acute Blocking in Fig. 3A). In the second protocol, AGA and HIV were applied simultaneously to the cultures (Co-application in Fig. 3A). Moreover, a more extensive analysis of TNTs in the presence of the GJ blockers (AGA or octanol) indicated that the synaptic contact induced by HIV infection was highly compromised, resulting in swelling of the synaptic contact (Fig. 3C) as compared to HIV infection induced TNT (Fig. 3B). Surprisingly, no significant differences in TNT formation were observed using either protocol (Fig. 3D). However, analysis of dye coupling to examine GJ communication between TNT connected cells using GJ channel blockers by either protocol (Acute and Co-application) totally abolished the diffusion of LY (Fig. 3E). Furthermore, quantification of the LY diffusion also demonstrated that GJ communication was blocked using both protocols (Fig. 3F). The vehicles, DMSO or ethanol, used to dissolve AGA and octanol, respectively, did not alter TNT formation, synaptic contacts, or dye coupling between TNT connected cells (data not shown). Thus, GJs are required for TNT-mediated intercellular communication via synaptic structures at the ends of the TNTs.

### TNTs and GJs at the tips of TNTs are required for effective HIV-replication and spreading

To determine whether TNT formation and GJs at the end of TNT processes play a role in the HIV life cycle in human macrophages, the protocols outlined in Fig. 3A were used to determine their impact on HIV replication and cell-to-cell spread of infection.

**Figure 4:**
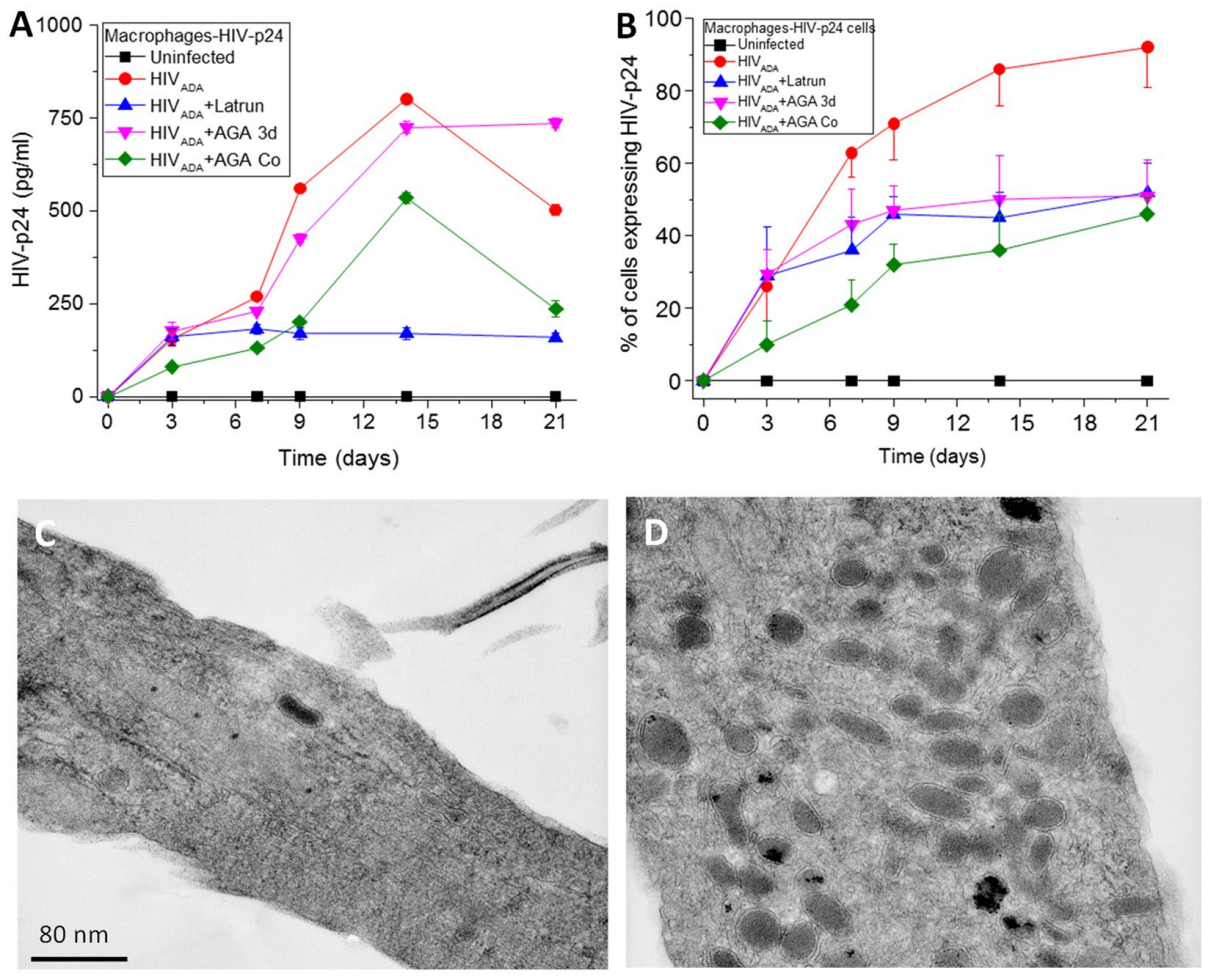
TNT formation and functional gap junctional communication are required for effective HIV-replication and HIV-spread. Macrophages were infected with HIV using the 2 protocols described in Fig. 3. (**A**) HIV-replication was determined by the amount of HIV released into the media by ELISA. Uninfected, HIV infected cultures, HIV-infection plus latrunculin (a TNT blocker, 1 nM), or HIV-infection plus co-application of the GJ blocker cultures indicated that blocking formation of TNT or gap junctions reduces release of the virus (all points, p ≤ 0.005, n = 3). The only no significant treatment was HIV-infection plus acute AGA may be due that we allowed 3 days of replication with the significant cell to cell spread of infection. (**B**) Quantification of the HIV-p24 positive cells in panel (**A**). All blockers reduced the spread of HIV-p24 as compared to HIV infection alone (all points, p ≤ 0.0001, n = 3). (**C** and **D**) Transmission electron microscopy (TEM) analysis of a perpendicular section of a TNT in HIV infected conditions. In both cases, no HIV virions were detected inside of the TNT. However, mitochondria were concentrated in areas where TNT are formed. Thus, the entire virion is not transferred by TNTs.

To measure HIV replication, HIV-p24 ELISA of the media was performed using both protocols. No HIV-p24 was detected in uninfected cultures (Fig. 4A, black line). Under conditions of HIV_ADA_ infection, viral replication was robust (Fig. 4A, HIV_ADA_, red line). Co-application of the GJ blocker resulted in a significant decrease in HIV replication at all time points tested (Fig. 4B p ≤ 0.001, green line). In contrast, acute application of the blocker did not affect replication (HIV_ADA_ + AGA, pink line, Fig. 4B) as compared to HIV infection alone. Furthermore, acute application of latrunculin (an F-actin blocker that inhibits TNT formation at 1 nM) at 24 h post-infection also resulted in a significant decrease in HIV replication after 7 days’ post-infection (p ≤ 0.0003, blue line in Fig. 4A). As indicated in Fig. 4B, the % of macrophages positive for HIV-p24 correlated with the time course of TNT formation (Fig. 1A,B). But interestingly, all treatments, latrunculin as well as acute and coapplication of AGA, reduced the numbers of HIV-p24 positive cells, suggesting that TNT containing GJ are essential for the cell to cell spread of the virus. In agreement, we detected a delay of 24 to 48 h for the subsequent release of virions into the extracellular space as compared to the percentage of HIV-p24 positive macrophages. Thus, viral infection, cell to cell spread, replication, and release stages can be separated according to the time course of TNT formation and communication. Thus, our data support the following time line, early HIV infection induces the formation of TNT to support cell to cell spread of the virus, subsequent replication and release of new virions into the extracellular space. Thus, both TNTs and GJs were required for effective HIV infection and subsequent replication to occur.

To further examine the role of TNTs and associated GJs in HIV spread and infectivity, the number of cells producing viral proteins, such as HIV-p24 were quantified to determine cell-to-cell spread of the virus. Depending on the donor, upon initial HIV infection of cultures of macrophages, between 10 and 35% of all cells become infected in the first round of infection, followed by cell-to-cell spread. Under all three treatment conditions (i.e., latrunculin to block TNT formation or acute or co-application of AGA or octanol to block GJ communication), the number of HIV-p24 expressing cells was significantly decreased after 3 days’ post-infection (p ≤ 0.004) (Fig. 4B). These data confirmed that TNTs and their associated GJs were required to spread infection (as measured by HIV-p24 levels) into neighboring cells.

To examine whether these TNTs directly mediate the spread of virions, transmission electron microscopy (TEM) was performed on these cell cultures, specifically focusing on the base of the TNT structure. As shown in Fig. 4C,D, TEM of TNT structures showed the presence of actin ‘cables’ and an increased presence of mitochondria aggregated within or near the TNT. Interestingly, there was no evidence of viral particles being present in the interior or exterior of the TNT. These results suggest that TNTs may not directly transfer the entire virion from infected to uninfected cells, but may sensitize the target cell to HIV-infection.

## Discussion

The results of this study have demonstrated the following. First, TNTs mediate long-range GJ communication. Second, Cx43 is mainly localized at the tips of the TNTs and forms functional GJ channels under conditions of HIV infection. Third, TNT numbers increase significantly in HIV-infected macrophages and mediate GJ communication between infected and uninfected cells. Fourth, these TNT/GJ connections are synapse-like. Fifth, functional GJs are not required for the formation and extension of the TNT process, but they are essential in the formation of the synaptic contact. Finally, GJs at the tip of the TNTs are essential to support efficient HIV replication and cell-to-cell spread. Together, these results establish that TNTs and GJs are essential during the process of amplification of infection and may be viral reactivation by mediating an effective cell-to-cell HIV infection.

The only known mechanism of direct cell-to-cell transfer of HIV is via specialized virological synapses^36,37^. HIV virological synapses between T cells and other cell types mediate direct translocation of large amounts of HIV antigens and proteins into the target cell to facilitate infection^38,39^. Several groups have calculated that such cell-to-cell transmission is up to 18,000-fold more efficient than for cell-free soluble virus^38,40^. This highly efficient mode of transmission of HIV shields the infectious agent from neutralizing antibodies and phagocytosis, allowing the infected cells to concentrate and transmit multiple viral genomes, reaching 10^2^–10^3^ copies in a single synapse^38,41^. The resulting increased HIV concentration could circumvent anti-retroviral treatments, thus providing a sanctuary for local replication, mutations, and resistance. Based on the results presented here, we propose that TNT/GJ processes can provide an additional route of rapid transfer of HIV proteins and viral mRNA or particles from HIV infected macrophages to uninfected cells, efficiently amplifying infection. Similar mechanisms of TNT-mediated cell-to-cell amplification of disease have been observed in cancer and neurodegenerative disorders (e.g., Alzheimer’s and Parkinson’s disease where aggregated proteins are transferred)^1,3,12,15,16,42,43^.

HIV has evolved to use Cx43 GJ communication to spread toxic signals from infected cells to connected uninfected cells^31,32,44^, as well as during the pathogenesis of HIV, including HIV entry, replication, reactivation,  transmigration of infected cells into tissues, inflammation, and the spread of apoptosis^7,44–46^. Although the underlying mechanism of TNT/GJ-mediated cell-to-cell viral spread is still unknown, a similar process in virological synapses involves the expression of key adhesion molecules, LFA-1, ICAM-1, and several integrins at the site of cell-to-cell contact^38,39^. Similar mechanisms of viral replication and amplification have been described for viruses like herpes viruses, hepatitis C, and Varicella Zoster^36,37^ where this mechanism has evolved to resist antibody neutralization and avoid the immune response.

Our results have identified at least two different stages of the HIV life cycle associated with TNT/GJ formation: the initial events of HIV infection and viral colonization and HIV reactivation. Both infection and reactivation stages require extremely efficient viral amplification that cannot be explained only by the cell-free virus because of the low viral load. For instance, during initial stages of HIV infection, the viral load transmitted to a mucosa or blood requires an efficient system of cell-to-cell spread to colonize the uninfected host^41,47–49^. This allows the virus to avoid detection and destruction by the immune system^50^. Thus, amplification of infection needs an exquisite mechanism to colonize the host immune system, and we propose that TNTs and GJs play a key role in this process. In the case of HIV reactivation^51–54^, several laboratories have demonstrated that in latently infected individuals only one cell is infected among 10^8^ to 10^12^ uninfected cells^51,55–58^. Thus, the process of viral reactivation also requires a highly efficient mechanism of viral spread that cannot be explained solely by soluble virus alone. Thus, alternative mechanisms of infection are likely involved, such as the TNT/GJ-dependent mechanism outlined in this paper.

Our data obtained by SEM and TEM support the presence of several types of TNTs that connect HIV infected and uninfected macrophages. Thus far, two different types of TNTs have been observed: those that are open-ended and allow direct communication between the cytoplasm of connected cells and others that are non-fused and characterized by synapse-like interactions. For example, lymphocytic TNTs were almost impermeable to calcium^59^, whereas the opposite was observed for TNTs in dendritic and THP-1 cells^60^, suggesting that different kinds of TNTs may be present in different cell types. TNTs observed in other biological systems allow the transport and transfer of mitochondria and vesicles into the target cell, suggesting that the internal pore size of the tube is large enough for the trafficking of these organelles (see review by^8,10,27^). We found that in macrophages Cx43 was mainly localized at the tips of the TNTs, which suggested that Cx43 played an important role in cell-to-cell communication over long distances (up to 200 µm). However, the presence of multiple processes associated with each TNT observed in this study supports the hypothesis that multiple types of TNTs are present, including the continuous types.

In conclusion, our data demonstrate that TNTs/GJs are a long-range intercellular communication system used by HIV-infected cells to spread infection to uninfected cells. Moreover, this mechanism helps amplify HIV infection by increasing the chances of small populations of HIV-infected macrophages to spread infection to a large number of uninfected cells. Thus, selectively blocking this TNT/GJ communication system has the potential to reduce or prevent viral infection and reactivation.

### Data availability

All data sets will be shared upon request.

### Ethical Approval and informed consent

All protocols were carried out in accordance with the guidelines and regulations of the NIH and Rutgers University.
